# Ohio Dental College

**Published:** 1868-03

**Authors:** 


### OHIO DENTAL COLLEGE.

The 22d annual commencement of the Ohio Dental College was held in the hall of the college building on the evening of the 4th of March.

The exercises of the occasion were introduced by the Rev. Henry D. Moore, by reading the scriptures, prayer, and some very interesting practical remarks on the great business and duty of life. After which the President of the Board of Trustees, Dr. G. W. Keely, delivered the annual address, and conferred the degree of D. D. S. upon the members of the senior class who had passed a satisfactory examination upon the various branches embraced in the course.

The following are the names of the graduates: R. J. Omahony, of Lexington, Kentucky; J. P. Holmes, Terry, Miss.; W. H. Thrift, Boonsboro, Iowa; R. N. Lawrence, Atlanta, Ill.; J. K. Morse, Ypsilanti, Mich.; J. C. Patchell, Hamilton, Ohio; B. F. Mills, Brooklyn, N. Y.; Wm. E. Tate, New London, Conn.; G. W. J. Shepherd, Cincinnati, Ohio.

The valedictory on behalf of the class was delivered by R. J. Omahony, for which see another page of this number.

The junior members of the class having passed a satisfactory examination upon the branches embraced in the junior year, were each presented with a certificate to that effect, by the Dean of the Faculty, which certificate admits them to the senior class without further examination.

The examination of the juniors upon the branches of their course was the same as that of the seniors upon the same branches, and was eminently satisfactory.

Names of juniors: J. L. Oldham, J. F. Dennis, Jas. S. Cassidy, Jas. Gamble, C. W. Barnes, J. T. Thompson, J. B. Alaxander, T. O. Paine, W. W. Switzer, M. N. Maguet, J. A. Young, E. C. Moore, C. H. Stephenson, C. R. Sabin, Chas. Penfield, A. Warner, Jr., H. K. Lathrop.

A large assemblage of the friends of the Institution and of the class was present, and were much interested in the exercises. Thus passed one of the most pleasant commencements ever held in the Institution. T.

